# Current NMR Techniques for Structure-Based Drug Discovery

**DOI:** 10.3390/molecules23010148

**Published:** 2018-01-12

**Authors:** Toshihiko Sugiki, Kyoko Furuita, Toshimichi Fujiwara, Chojiro Kojima

**Affiliations:** 1 Institute for Protein Research, Osaka University, Osaka 565-0871, Japan; sugiki@protein.osaka-u.ac.jp (T.S.); k-furuit@protein.osaka-u.ac.jp (K.F.); tfjwr@protein.osaka-u.ac.jp (T.F.); 2 Graduate School of Engineering, Yokohama National University, Yokohama 240-8501, Japan

**Keywords:** nuclear magnetic resonance (NMR), NMR-based fragment screening, NMR-based lead optimization, ligand-based NMR, protein-based NMR, fluorine-19 (^19^F) NMR, site-specific isotope labeling, protein-protein interaction (PPI) breaker/stabilizer

## Abstract

A variety of nuclear magnetic resonance (NMR) applications have been developed for structure-based drug discovery (SBDD). NMR provides many advantages over other methods, such as the ability to directly observe chemical compounds and target biomolecules, and to be used for ligand-based and protein-based approaches. NMR can also provide important information about the interactions in a protein-ligand complex, such as structure, dynamics, and affinity, even when the interaction is too weak to be detected by ELISA or fluorescence resonance energy transfer (FRET)-based high-throughput screening (HTS) or to be crystalized. In this study, we reviewed current NMR techniques. We focused on recent progress in NMR measurement and sample preparation techniques that have expanded the potential of NMR-based SBDD, such as fluorine NMR (^19^F-NMR) screening, structure modeling of weak complexes, and site-specific isotope labeling of challenging targets.

## 1. Introduction

In many case, the first step in the development of new pharmaceuticals is the discovery of new molecules from a library comprising a myriad of chemical compounds and natural products that show disease preventive actions, by performing a large number of in vitro and in vivo screening experiments [1]. The therapeutic effects are obtained by either inhibiting or activating molecular function. For successful drug discovery or development, directly identifying biomolecules participating in disease initiation and progression, and determining their intermolecular interaction mode at an atomic resolution, are important. Structure-based (or assisted) rational drug development (SBDD), using X-ray crystallography, computational molecular modeling/docking, and nuclear magnetic resonance (NMR) methods, are powerful and straightforward approaches. For example, X-ray and NMR were used to discover a potent and selective allosteric ABL1 tyrosine kinase inhibitor that is undergoing clinical development testing in patients with leukaemia [2], and NMR-based screening and SBDD were used to discover a BCL-2 inhibitor that is undergoing clinical trials in lymphomas, leukemia, and myeloma [3].

**Figure 1:**
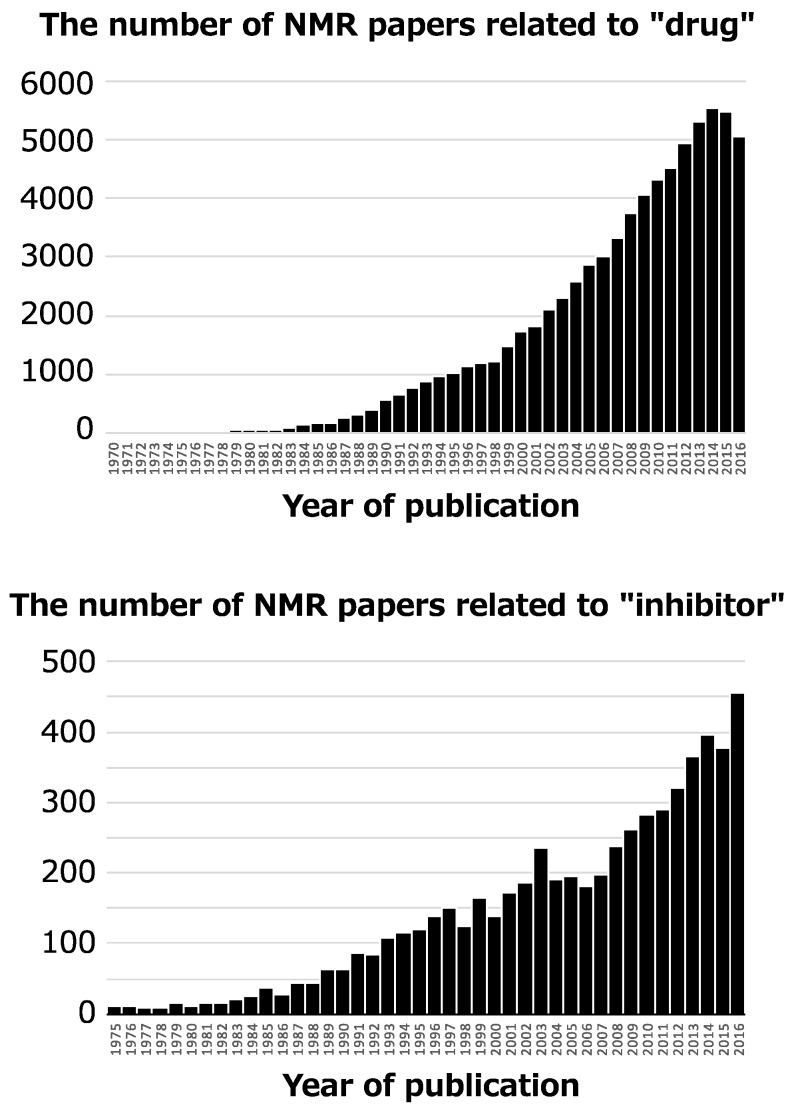
The number of NMR papers with the key words “drug” published between 1970 and 2016 (**Top**) and “inhibitor” between 1975 and 2016 (**Bottom**). These numbers are obtained from PubMed search (https://www.ncbi.nlm.nih.gov/pubmed).

NMR has been used for an enormous number of pharmaceutical studies since the 1970s, and the number of NMR papers with the key words “drug” and “inhibitor” is increasing (Figure 1). Typical NMR application is discovery of specific inhibitor and its binding mode analysis [4], and remarkable one is discovery of allosteric regulator by NMR-based screening and its conformational analysis [5,6]. For such studies, many NMR methods have been developed due to the advantages of NMR [7]. These advantages include: both chemical compounds and biomolecules give NMR signals, the binding mode between chemical compounds and biomolecules, such as the tertiary structures, conformational changes, and interaction interface, can be determined at an atomic resolution, and NMR performs well for weak intermolecular interactions with dissociation constant (*K_d_*) in the μM~mM range.

Due to developments in data acquisition and processing hardware and methodologies, high-resolution NMR spectra can be observed with high sensitivity and reproducibility. Fast NMR data acquisition has led to remarkable improvements in the throughput of high-resolution and sensitive NMR methodologies, and has created a new avenue for fragment-based drug discovery and development (FBDD) for identifying new fragments.

Fragment-based screening strategies have the potential to discover new small binders, which have novel pharmacophores scaffolds. Chemical linking and growth of the small binders, based on structural information of the target pocket of the protein, is a rational strategy used to generate new compounds. An ideal compound completely occupies the target pocket with higher affinity and selectivity [8]. In general, the fragment is a small molecule, typically less than 300 Da [9], and the interaction between the fragment and target protein tends to be too weak to be detected by sensitive biochemical and biophysical assays, such as ELISA and fluorescence resonance energy transfer (FRET)-based high-throughput screening (HTS), where the high concentration of molecule hides the response. X-ray crystallography is used to detect such weak interactions although these ligands may not always co-crystalized. In FBDD, NMR can be applied to both screening and hit-to-lead optimization [10]. NMR-based SBDD approaches could lead to the discovery of unique seeds for next-generation drugs that show effective pharmaceutical action, driven by new mechanisms suppressing drug resistance.

## 2. NMR Spectroscopy Aimed at Drug Discovery-Ligand-Based and Protein-Based Approaches

Pharmaceutical NMR methodologies can be divided into two major categories: ligand-based and protein-based [10,11]. The ligand-based approach has the following features: (1) one-dimensional (1D) hydrogen (^1^H) or fluorine (^19^F) NMR experiments are used; (2) isotope labeling of the target protein is unnecessary; (3) rapid and sensitive NMR measurement is possible with lower protein concentrations, generally 5–50 μM. The total amount of sample can be reduced by using a target protein-immobilized NMR screening (TINS) method [12]; (4) no upper limit on the size of the target protein exists; (5) sample purity conditions are more relaxed, or less strict, provided that contaminants and impurities present in the solution do not interfere with the stability and function of the target protein and fragment.

In the protein-based approach, ^1^H-^15^N and ^1^H-^13^C heteronuclear single quantum correlation (HSQC) spectra are measured for uniformly ^15^N- and ^13^C-labeled proteins, respectively, in the absence or presence of ligands. The ligand binding site of the target protein is identified by HSQC chemical shift perturbation and signal broadening induced by ligand binding [13]. This approach can be applied to extremely low affinity interactions, with *K_d_* in the ~mM range. ^15^N-labeled protein (~0.1 mM, depends on the size of the protein) is easy and inexpensive to prepare. ^1^H-^15^N HSQC spectra are sufficiently sensitive to monitor structural changes and ligand binding, and are widely used for the fingerprinting of proteins.

In the early stage of SBDD, a ligand-based approach is useful for the screening of hit ligands. Protein-based approaches are useful for hit validation, based on affinity and binding modes, and for the selection of hit ligands from false-positive and non-specific binders. At the hit-to-lead optimization stage, both approaches are useful. For example, a ligand-based approach can identify pharmacophores using competitive ligands, and a protein-based approach is useful for structure determinations of protein-ligand complexes [14,15].

### 2.1. Ligand-Based NMR Approaches for SBDD

Ligand-based NMR approaches have limitations with respect to the exchange rate between the target protein and ligand compound. With increasing affinity and decreasing ligand dissociation speed from the target protein (off rate of inter-molecular interaction, *k*_off_), the detection of target protein-ligand binding is problematic since ligand signals do not reflect the protein-bound form, due to slow exchange. In general, an appropriate range for the dissociation constant between the target protein and ligand for successful ligand-based NMR approaches is about 1 mM to 0.1 μM depending on the exchange rate [16]. Therefor the ligand-based approach is difficult to apply to strong binders without advanced NMR techniques [17]. Moreover, an excess concentration of ligand is used compared to the target protein to ensure fast exchange. Under these conditions, the ligand NMR signal of the free state reflects a trace memory recorded on the ligand magnetization when the ligand remains on the target protein [16].

**Figure 2:**
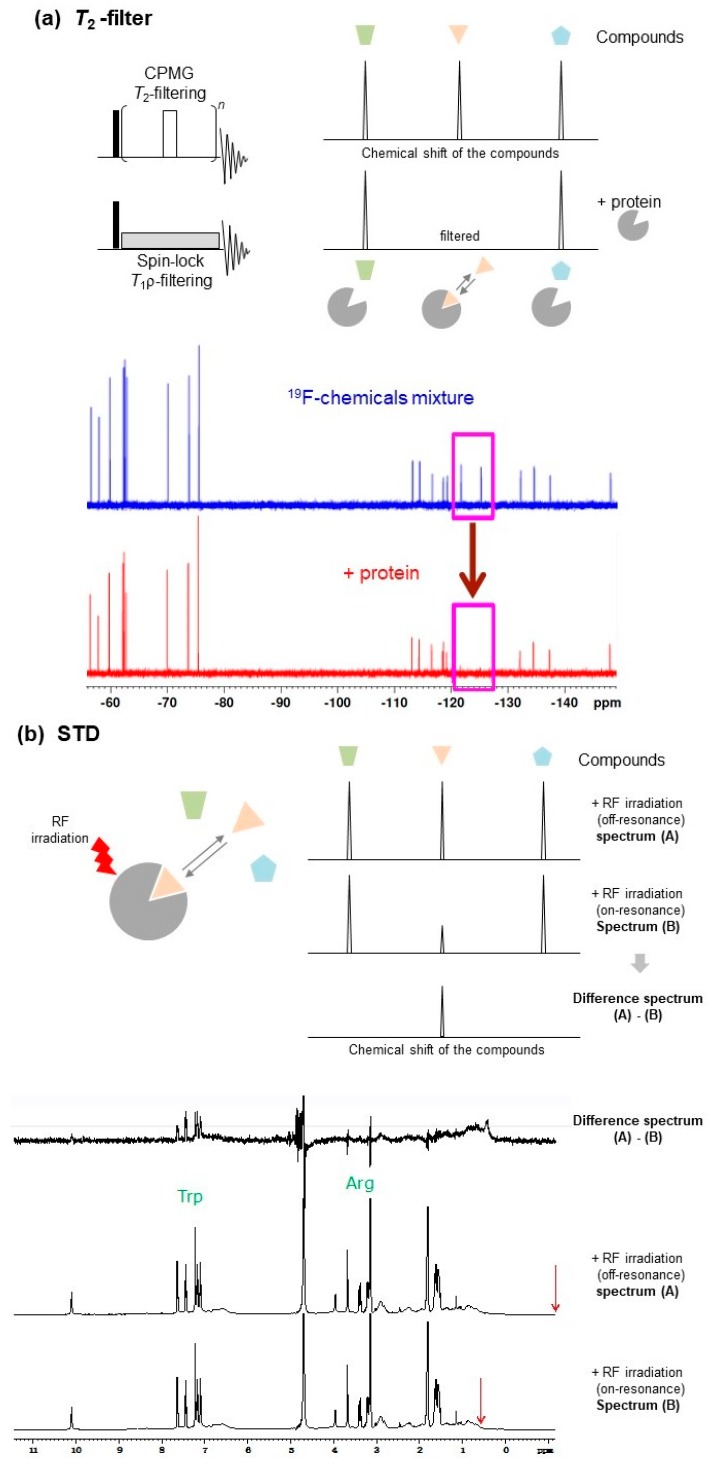

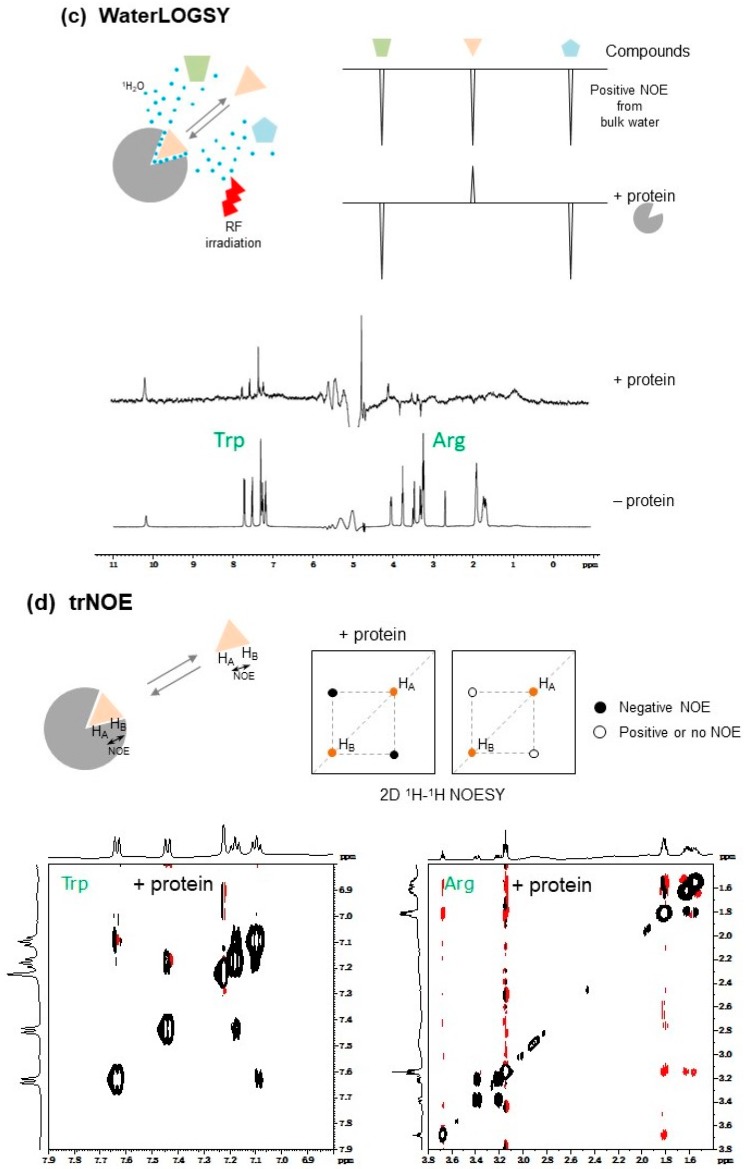

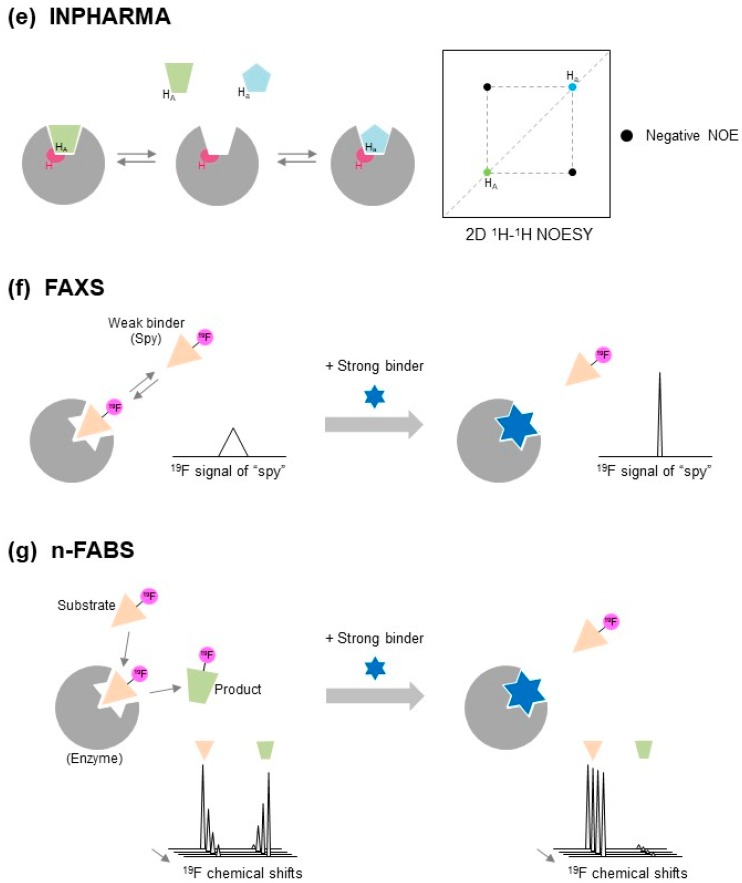
Ligand-based nuclear magnetic resonance (NMR) approach for structure-based drug discovery (SBDD) studies. The trapezoid, triangle, and pentagon, colored in green, pink, and light blue, respectively, indicate different compounds. The grey circle lacking the wedge shape indicates target protein. NMR spectra are taken from textbook used in “Pharmaceutical NMR Lecture Series in Osaka” held at Institute for Protein Research, Osaka University in 2012: (**a**) T_2_-filter. (Left) NMR pulse schemes for T_2_- and T_1ρ_-filtering experiments. The black and white bars indicate 90° and 180° pulses, respectively. (Right) The signal intensity of hit ligand (triangle colored in pink) is significantly attenuated by Carr-Purcell Meiboom-Gill (CPMG) or spin-lock pulse due to interaction with protein. (Bottom) ^19^F T_2_-filter spectra of ^19^F-chemicals (red) with and (blue) without protein; (**b**) Saturation transfer difference (STD). (Left) Pink triangle indicates hit ligand. NMR signal of the target protein is selectively saturated by radio frequency (RF) irradiation. This saturation is specifically transferred to the hit ligand (pink triangle). (Right) The signal intensity of the hit compound is significantly modulated by saturation due to interacting with protein. When the difference spectrum between the on-resonance and off-resonance saturation is observed, NMR signals from hit compounds are easily identified. (Bottom) STD spectra of the solution mixture containing l-tryptophan (as the binder), l-arginine (as non-binder), and BSA (protein); (**c**) Water-ligand observed via gradient spectroscopy (WaterLOGSY). (Left) Pink triangle and light-blue dots indicate hit ligand and water molecules, respectively. NMR signal of water is selectively saturated by RF irradiation. This saturation is specifically transferred to hit ligand (pink triangle) as intermolecular nuclear Overhauser effect (NOE) through protein-ligand complex formation. (Right) The signal of the hit ligand is reverted due to interaction with protein. (Bottom) WaterLOGSY spectra of the solution mixture containing l-tryptophan (as the binder), l-arginine (as non-binder), and BSA (protein); (**d**) Transferred NOE (trNOE). (Top Left) Pink triangle indicates hit ligand. Intra-ligand ^1^H-^1^H NOE is significantly enhanced when the hit ligand is located on the target protein. (Top Right) The open and filled circles are negative and positive/no peaks, respectively. The orange and black circles indicate diagonal and cross peaks, respectively. The sign of the NOE cross-peaks of the hit ligand is negative, due to interaction with protein and increase in rotational correlation time (τ_c_) of the ligand. (Bottom Left) trNOE spectra of the solution mixture containing l-tryptophan (as the binder) and BSA (protein), and (Bottom Right) l-arginine (as non-binder), and BSA (protein). The black and red lines are positive and negative peaks, respectively; (**e**) Interligand NOEs for Pharmacophore Mapping (INPHARMA) method. (Left) Green trapezoid and light-blue pentagon indicate two different hit ligands binding to the same site. The inter-molecular NOE between competitive binding ligands increases at the ligand binding site (colored in magenta) on the target protein. (Right) The light-blue, green, and black-filled circles indicate diagonal peaks of competitive binding ligands (green trapezoid and light-blue pentagon), and inter-ligand negative NOE cross-peaks, respectively; (**f**) Fluorine chemical shift anisotropy and exchange for screening (FAXS). Pink triangle and blue hexagram indicate ^19^F-labeled weak binder (spy molecule) and competitive strong binder, respectively. When the competitive strong binder is mixed with the target protein in the presence of the ^19^F-labeled weak binder, the weak binder is released and its fluorine NMR (^19^F-NMR) signal intensity is recovered; (**g**) n-fluorine atoms for biochemical screening (n-FABS). Pink triangle, green trapezoid, and blue hexagram indicate ^19^F-labeled substrate, ^19^F-labeled product, and competitive strong binder, respectively. When the competitive strong binder is mixed with the target protein in the presence of the ^19^F-labeled substrate, the substrate is released and its ^19^F-NMR signal becomes time-independent without enzymatic reaction.

For ligand-based NMR experiments, a solution mixture including l-tryptophan (e.g., 1 mM, as a binder), sucrose (e.g., 1 mM, as non-binder), and bovine serum albumin (BSA) (e.g., 0.1 mM) has been recommended as a standard sample [18]. This sample is useful to test pulse sequences (Figure 2). However, experimental parameters and conditions for the desired NMR measurements should be optimized for each protein. Most ligand-based NMR methods do not require protein isotope labeling.

As shown below, many techniques have been reported in ligand-based NMR approaches. However, each technique has advantages and disadvantages. In an effort to obtain reliable screening results, validating hit compounds using a variety of different techniques is preferable [18].

#### 2.1.1. T_2_-Filter

Protein-ligand interactions can be investigated by examining the increase in the transverse relaxation rate of the ligand NMR signal. The transverse relaxation rate is accelerated by the decreased rotational diffusion rate and increased rotational correlation time due to protein-ligand binding [19]. In T_2_- and T_1ρ_-filter [20,21,22] experiments (Figure 2a), protein-ligand binding was detected by the decrease in resonance intensity of the ligand NMR signals. This intensity reduction is mainly from the apparent line-broadening induced by the exchange process between the free and bound states.

#### 2.1.2. Paramagnetic NMR

**Figure 3:**
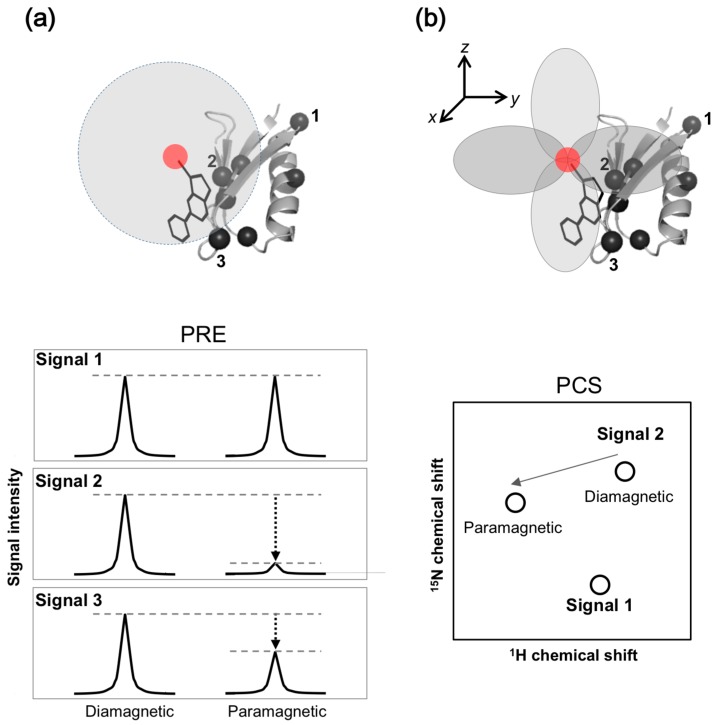
Paramagnetic effects utilized for protein-based NMR approaches for SBDD studies. Effects of (**a**) paramagnetic relaxation enhancement and (**b**) pseudo-contact shift are illustrated. Paramagnetic center denoted with red sphere is chemically immobilized on the ligand or protein. Black spheres on the protein indicate position of isotopically-labeled moieties. Numbers correspond to each NMR signal. (**a**) The changes of signal intensity of protein are induced by paramagnetic effect depending on the distance between paramagnetic center and position of isotopically-labeled moieties. (**b**) The changes of chemical shift of protein depend on the distance between paramagnetic center and position of isotopically-labeled moieties, and at an angle with magnetic field.

Paramagnetic NMR, such as paramagnetic relaxation enhancement (PRE), increases the transverse relaxation rate and can be used as an alternative to the T_2_-filter method. In PRE, the transverse relaxation rate is accelerated by dipolar interactions with unpaired electrons from spin-label or paramagnetic metal ions. In paramagnetic NMR experiments, a spin-label or paramagnetic metal ion is attached to the protein. Protein-ligand binding is detected by the decrease in signal intensity of the drug due to PRE, resulting from the unpaired electron immobilized on the protein (Figure 3a).

With the pseudo-contact shift (PCS) approach, the other paramagnetic NMR approach, a lanthanide ion is attached to the protein. Protein-ligand binding is detected by the chemical shift change induced by PCS from the lanthanide ion attached to the protein (Figure 3b). PCS can be combined with PRE, and detected by ^1^H and ^19^F signals derived from the ligands [23]. For drug screening, the Spin Labels Attached to Protein Side chain as Tool to identify Interacting Compounds (SLAPSTIC) method for measuring spin-label-induced T_1ρ_ relaxation enhancement has been reported [24]. These approaches have also been applied to FBDD [25].

The paramagnetic NMR approach is dependent on the distance (*r*^−6^ and *r*^−3^ for PRE and PCS, respectively) between the observing nucleus and the paramagnetic center. Distance information provided by paramagnetic NMR, up to 30 and 40 Å for PRE and PCS, respectively, is significantly longer than inter-proton nuclear Overhauser effect (NOE) of up to 6 Å. Additionally, PCS provides angular information between the observing nucleus and paramagnetic center. This information is widely used to investigate inter-molecular interactions, especially for ephemeral or short-lived but biologically significant conformers, such as transient “intermediates” or “activated” forms of target proteins. In some cases, the tertiary structure of a protein and protein-ligand complexes were determined [26,27,28,29].

For paramagnetic NMR, spin-labeled or paramagnetic metal ion-tagged proteins should be prepared (Section 3.3). Conversely, chemical compounds can be spin-labeled or tagged with a paramagnetic metal ion. In this case, protein-ligand binding is detected by the PRE- or PCS-induced signal disturbance of the target protein due to PRE from the paramagnetic center of the drug [11,30]. Notably, the spin-labeling technique is particularly suitable for nucleic acids since the site-specific chemical modification of nucleic acids is technically straightforward [31].

#### 2.1.3. Diffusion Ordered Spectroscopy (DOSY)

In addition to the rotational diffusion-based techniques, translational diffusion-based NMR experiments, such as DOSY, have also been used for drug screening [20,32]. Although the translational diffusion time is less sensitive to the molecular weight of a sample compared to the rotational correlation time [33], small lined and protein are easily distinguished. The translational diffusion-based approach can be used for pulse schemes to edit coherences, such as COSY-DOSY, TOCSY-DOSY, HSQC-DOSY, NOESY-DOSY and STD-DOSY [34,35,36]. These experiments are quite powerful for selective observation of NMR signals from bound-state ligand eliminating signals from free-state ligand and vice versa [34,35,36]. NOE-pumping pulse techniques [37,38] are useful for observing NOE cross-peaks of bound-state ligands, with higher sensitivity and selectivity by filtering signals derived from the free-state ligand before the NOE mixing time.

#### 2.1.4. NOE-Based Methods

When a small ligand interacts with a protein, the apparent molecular weight (rotational correlation time, τ_c_) of the ligand in the bound state increases depending on the molecular weight of the target protein. For example, when the molecular weight of a ligand and a protein is 300 Da (τ_c_ = ca. 0.2 ns) and 30 kDa (τ_c_ = ca. 20 ns), respectively, the intra-ligand NOE becomes 20 times stronger if the ligand interacted with the protein [36]. Therefore, protein-ligand interactions can be assessed by NOE-based methods, such as saturation-transfer difference (STD) [39,40], SOS-NMR [41], WaterLOGSY and its related methods [42,43], transferred NOE (trNOE) [44], INPHARMA [45,46], and inter-ligand NOE (ILOE) [47]. These methods use NOE and magnetization transfer from the target protein or other molecules, such as bulk water and ligand, to ligands through dipole–dipole interactions (Figure 2). These dipole–dipole interactions depend on the molecular weight, therefore they do not perform well with proteins of low molecular weight.

Slower molecular tumbling, such as with ligands binding to protein, yields negative NOE cross-peaks, which is the same sign as the diagonal peaks [48], although the NOE effect in free ligand is positive (opposite sign to diagonal peaks) (Figure 2b–e). This sign conversion occurs around 1 kDa [48]. Under rapid exchange between free and bound states, such as with the trNOE and INPHARMA methods, a negative NOE is observed in the NOESY spectrum of free ligand [49].

These NOE-based NMR approaches have the potential for simple screening of binders and for rough epitope estimation, group epitope mapping (GEM), and characterization of the target protein-binding mode of ligands, such as conformation and orientation at atomic resolution [18,39,44].

#### 2.1.5. STD

In STD experiments (Figure 2b), the proton resonance of the target protein (e.g., methyl ^1^H signals, the chemical shifts of which are located far from the ligand signals, typically ~−0.5 ppm) is selectively saturated by irradiation, avoiding direct saturation of the ligand signals, and then the ^1^H-NMR data of the ligands are immediately collected. When a ligand binds to a target protein, the saturation of the proton magnetization on the target protein is transferred to the ligand through dipole–dipole interactions, and the ^1^H signal intensity of the free ligand is modulated under fast exchange conditions between the free and bound states.

STD is typically observed when the dissociation rate *k*_off_ is greater than the longitudinal relaxation rate 1/T_1_ of the free ligand. The *k*_off_ values vary to some extent with changing experimental conditions. STD parameters, such as power of saturation pulse, saturation period (typically 1–2 s, varying depending on the molecular weight of the protein), and frequency center of irradiation pulse for saturation, should be optimized using only the ligand in the absence of protein to verify that conditions will not directly lead to saturated signals of the ligand. Following this, buffer conditions and the concentration of protein and ligand (the typical concentrations of protein and ligand are 2.0–20 μM and 0.2–2.0 mM, respectively, the ranges of which may depend on solubility of the chemical compounds in water or available amounts of both interacting partners) should be finely tuned to obtain sufficient and significant binder signal intensity loss, while avoiding false-positives due to protein–protein, protein–ligand, or inter-ligand non-specific associations [18].

With STD, the target protein-binding mode of ligands can be determined even if the affinity is weak. For example, the Complete Relaxation and Conformational Exchange Matrix analysis of Saturation Transfer (CORCEMA-ST) program validates tertiary structure models of protein-ligand complexes utilizing STD data [50,51].

#### 2.1.6. SOS-NMR

The SOS-NMR method (a definition of the acronym SOS-NMR is not provided in the original paper) is based on STD and is characterized by the use of a target protein that is site-specifically ^1^H-labeled with ^2^H-substitution (deuteration) of other undesired non-labile protons [41]. Ligand bound to the ^1^H-labeled site of the target protein can be selectively detected by this method, while excluding ligand bound to undesired binding sites. The SOS-NMR method provides structural information about the relative orientation of the ligand with target protein in a bound state, by performing a series of experiments with varied ^1^H-labeling of the target protein site. However, the sensitivity of this method is generally lower than that of STD since dipole–dipole interactions are limited due to low ^1^H density on the target protein.

#### 2.1.7. WaterLOGSY

The water-ligand observed via gradient spectroscopy (WaterLOGSY) [42] method, and its related solvent accessibility, ligand binding, and mapping of ligand orientation by NMR spectroscopy (SALMON) technique [43], are water-saturating STD-like methods. These methods use the characteristic of the saturation transfer efficiency being higher for hydration water of a protein compared to free bulk water (Figure 2c). Mainly due to the difference in τ_c_ of the water, the sign of the NOE cross-peaks between the water and ligand can be altered; positive NOE cross-peaks can be observed between the free bulk water and ligand, and negative NOE cross-peaks can be observed between protein-bound hydration water and ligand (Figure 2c).

#### 2.1.8. trNOE

The trNOE method is a powerful approach for the screening of chemical libraries using the sign inversion of the intra-ligand NOE cross-peaks. In trNOE experiments (Figure 2d), the typical NOE mixing time of a trNOE measurement is 200–600 ms and should be optimized by considering the sample temperature, viscosity of the sample solution, and molecular weight of the protein [48]. trNOE experiments provide structural information of the ligand in the bound state and insight into any conformational changes of the ligand induced by interaction with a protein. When the ligand has no preferential structure in the free state and the structure appears exclusively in the bound state, there is no change of the sign of NOE. This trNOE information can provide insights for use in hit-to-lead optimization studies [49].

#### 2.1.9. INPHARMA and ILOE

Interligand NOEs for Pharmacophore Mapping (INPHARMA) [45,46] and Inter-ligand nuclear Overhauser effect (ILOE) [47] methods are based on ligand-to-ligand NOEs via target protein (Figure 2e). With the INPHARMA method, inter-ligand NOEs between two ligands, that competitively bind to the same binding site on the target protein, are measured. On the other hand, the structure-activity relationship (SAR) with the ILOE approach uses target protein-mediated ligand-ligand NOEs (ILOEs), and identifies two individual ligands that bind to the target protein simultaneously in close proximity. Both ligands do not necessarily bind to the same binding site [47]. The ILOE method requires longer NOE mixing times, typically 600–800 ms, compared to the INPHARMA method which typically requires 50–100 ms [45,49].

The INPHARMA and ILOE methods are unique in that these approaches allow the identification of new variants of known binders for a specific binding pocket on a target protein, and to generate new “known drug—novel fragment hybrid compounds” showing higher affinity by optimizing drugs through chemical linking and growing using identified binders, known as SAR-by-NMR and fragment-growing [10,49].

The INPHARMA method determines the relative orientation of two individual ligands if the molecular orientation of one ligand on the target protein is already known [45,46]. By measuring the INPHARMA spectrum of two ligands, even if the conformation or orientation of both ligands are unknown, the correct binding modes of the two ligands and their pharmacophore can be determined by combined use of docking analyses and back-calculation of the INPHARMA spectrum, using the CORCEMA approach [49,52,53].

#### 2.1.10. ^19^F-NMR

The ^19^F nucleus, which is a 100% naturally abundant fluorine NMR-visible isotope, is absent in biomolecules, and its NMR sensitivity is comparable to that of ^1^H (~83%). ^19^F-NMR has a wide chemical shift range and is sensitive in terms of reflecting local chemical environments other than ^1^H. Transverse relaxation of the ^19^F spin is dominated by chemical shift anisotropy (CSA), even at a lower magnetic field, such as 500 MHz [36]. The CSA-dominated transverse spin relaxation is sensitive to τ_c_ [36], so ^19^F-NMR is sensitive to τ_c_ of the sample molecules and gives sharp signal for small molecules. ^19^F-NMR is also a sensitive technique used for the examination of interactions between protein and ^19^F-containing ligand, and can be applied in cases involving short-lived bound states that include only about 1–3% of the total protein/ligand population [36]. Recently, ^19^F-NMR has been drastically improved in terms of sensitivity and throughput due to the development of ^19^F-tuned cryogenic probes and optimization of pulse sequences and parameters.

Based on these advantages, ^19^F-NMR has been widely used as a tool for ligand-based and protein-based NMR approaches in pharmaceutical studies [54]. For example, although a typical ligand-based approach used ^1^H-NMR, it is now possible to do accomplish the same by ^19^F-NMR using ^19^F-labeled compounds [55]. Three kinds of STD experiments, ^1^H saturated and ^19^F observed, ^19^F saturated and ^1^H observed, and ^19^F saturated and ^19^F observed, have been used to investigate the interaction between perfluorinated aromatic xenobiotics and dissolved humic acids, or the interaction between proteins and fluorine-containing ligands [56,57]. Dalvit and co-workers developed ligand-based screening methods using ^19^F as an NMR reporter, referred to as FAXS (Fluorine chemical shift anisotropy and exchange for screening) and n-FABS (n-fluorine atoms for biochemical screening) [16,58,59] (Figure 2). In protein-based ^19^F-NMR-based screening approaches, PrOF (Protein-observed ^19^F) NMR has been reported [60].

#### 2.1.11. FAXS

The FAXS method is an NMR-based ligand binding-competition approach used to explore new binders of target proteins by employing ^19^F-containing “spy” molecules with weak affinity (Figure 2f). When the spy molecule occupies the ligand-binding pocket of the target protein, its ^19^F signal will be broadened. If the spy molecule is replaced with a higher-affinity ligand in a competitive manner, this is detected by a restoration in the line width of the ^19^F signal of the spy molecules [58]. When the binding constant of the spy molecule is known, the affinity of the hit compound can be determined even if the hit compound binds too strongly to detect the free-state NMR signal. This competition experiment with a weak binder as “spy” is particularly important. When there is a strong binder in the cocktail, even the sophisticated direct methods (such as waterLOGSY, STD, …) fail to find other binders. This is because the protein is at low concentration, the strong binder will only be weakened by some %, and the other molecules will not interact anymore. Therefor the ligand-based approach except for FAXS is difficult to apply to strong binders in general, and the protein-based approach which is sensitive to strong binder is recommended.

#### 2.1.12. n-FABS

The n-FABS method is an NMR-based fragment screening approach that uses the enzymatic activity of the target protein (Figure 2g). In this approach, an enzymatic activity assay is performed typically using trifluoromethyl (CF_3_)-tagged known substrates of the protein enzyme. As a result, the distinct chemical shifts of the ^19^F signals of CF_3_ can be observed for the CF_3_-tagged substrate and products (Figure 2g). When high-affinity ligand co-exists with CF_3_-tagged substrates, the enzymatic reaction can be satisfied, leading to a loss in ^19^F chemical shifts derived from the products [16,59]. The n-FABS method is a powerful approach that can identify a new ligand that binds to the active center of a target protein enzyme.

#### 2.1.13. ^19^F-Chemical Libraries

Fluorination of compounds is a strategy used to increase the drug potential of compounds, since the presence of fluorine atoms in the compounds significantly influences structural and physicochemical characters, such as electronic and steric profiles, lipophilicity and solubility, metabolic stability, target protein recognition mode, and pharmacokinetic properties [54].

In ^19^F-NMR, a ^19^F-chemical library is used at the initial drug screening stage following a lead-optimization step [22]. When using a ^19^F-chemical library and ^19^F-NMR, each of the ^19^F-chemicals is easily identified on ^19^F-NMR spectra without signal overlap and water-suppression [61]. The interaction between the chemicals in the library and target protein can be readily and unambiguously identified by evaluating signal intensity reduction and chemical shift perturbation (CSP) of ^19^F-NMR signals in the absence or presence of target protein.

In general, experimental designs aim at efficient, prompt, and complete screening, with thousands of ^19^F-chemicals being divided into mixtures, typically consisting of 10–20 fragments, and hit screening is sequentially executed for each mixture to improve its throughput [62]. Hit candidates are identified by ^19^F-NMR signal changes from 10 to 20 merged peaks. Therefore, carefully preparing each chemical cocktail is important to ensure that all individual ^19^F signals of the 10–20 compounds in the mixture can be observed without overlap in the one-dimensional (1D) ^19^F spectrum. When a 1D NMR experiment per cocktail takes 20–30 min to complete, screening 2000 fragments (100 cocktails) would take between 48 and 50 h to complete.

All compounds in the ^19^F-chemical library should contain fluorine atoms, which may limit the library size and structural variety of the compounds. Assessment of the quality control of the chemicals, prior to initiating the NMR-based screening experiments, significantly influences the results [16,62]. Stock solution of the chemicals, typically dissolved in dimethyl sulfoxide (DMSO-*d*_6_) and the concentration of fragments is adjusted to 40–100 mM, is diluted with aqueous buffer to 40–100 μM. A quality check of the chemicals using NMR is important for assessing the solubility of each chemical against aqueous solvent, and for identifying and quantifying unexpected impurities or contaminants.

### 2.2. Protein-Based NMR SBDD Approaches

Protein-based NMR approaches are powerful methods to investigate protein–protein and protein–ligand interactions at atomic resolution, but not for drug screening due to the following limitations: (1) an appropriate isotope-labeled target protein is necessary; (2) milligram quantities of isotope-labeled protein, typically 20–200 μM, are required in a soluble form. This concentration is 10-fold higher than those that used in ligand-based NMR approaches, which are typically 2–20 μM; (3) the molecular weight of the protein is limited, typically less than 30 kDa, since the broadening and overlapping of signals becomes severe with increasing molecular weight; (4) the collection of high quality NMR spectra requires optimization of the sample and NMR measurement conditions by iterative and laborious test experiments; (5) a compound cocktail cannot be used without an additional deconvolution step. Further details on protein-based NMR methods are available in the literature [10,18,36].

When hit compounds are successfully obtained by screening, hit validation is required before SAR analyses and structure-guided hit-to-lead optimization. Protein-based NMR approaches are useful for hit validation with *K_d_* determination as well as pharmacophore estimation, fragment exploring, and rational hit-to-lead optimization. This approach is typically used for SAR analyses using the NMR technique [63]. Pharmaceutically non-optimized fragments, that possess diverse structures and engage in various binding sites, are chemically linked and grown or extended to fit adjacent druggable pockets on target proteins [11,64,65,66,67,68].

#### Protein–Protein Interaction (PPI)

Therapeutically relevant PPIs recently became a target for drugs. In fact, many PPI inhibitors have been developed and some are in the preclinical trial step [69]. Protein-based NMR experiments provide straightforward guidelines for the development of PPI modulators [65,70,71,72,73].

## 3. Isotope Labeling of Target Proteins for Drug Discovery by Protein-Based NMR

Isotope labeling of a recombinant protein of interest can be accomplished by heterologous protein expression systems, using living host cells such as *Escherichia coli* and yeast, as living bioreactors of protein over-expression using ^13^C-enriched sugars and/or ^15^N-enriched ammonium salts as carbon and nitrogen sources, respectively, as previously reviewed [10,74].

Uniform ^13^C and/or ^15^N labeling is necessary to assign NMR resonances of a target protein by performing traditional two-, three-, or higher-dimensional NMR measurements [10,74]. However, the difficulty of resonance assignment increases with increasing molecular weight and decreasing molecular tumbling speed, since these factors cause signal degeneration and line broadening. Especially the resonance assignments of α-helical transmembrane proteins and intrinsically disordered proteins are difficult, because their chemical shifts are generally less dispersed [75]. Therefore, amino acid-selective and site-specific isotope incorporation, and its related NMR measurement, are widely used as shown below.

### 3.1. Amino Acid-Selective ^13^C/^15^N Labeling and Unlabeling

Amino acid-selective isotope enrichment of a target protein is a useful alternative strategy to uniform isotope labeling, especially if difficulties occur in the preparation of sufficient amounts of uniformly ^13^C/^15^N-labeled target proteins and/or the measurement of sufficient quality triple-resonance spectra, due to limitations in target protein solubility and linewidth.

Amino acid-selective isotope labeling of tryptophan and arginine residue side chains is useful as a NMR detection probe, since these residues can be frequently identified in hot spots of the PPI interface [76,77]. Typically, the desired isotope-enriched amino acids are incorporated within the recombinant protein of interest by supplying isotope-enriched amino acids with other amino acids in unlabeled form in the cell cultivation medium or cell-free reaction solution.

Similarly, amino acid-selective unlabeling of desired heterologous protein is useful in a uniformly isotope-enriched background, called inverse labeling [78,79,80]. By combinatorial preparation of selective amino acid isotope-labeled/unlabeled protein samples, assignment of ^1^H-^15^N signals is possible without traditional triple-resonance NMR measurements [81].

### 3.2. Fractional and Site-Specific Isotope Labeling

In addition to selective amino acid labeling, fractional and site-specific isotope incorporation is powerful in simplifying NMR spectra toward pin-point observation of desired NMR signals (Figure 3). This approach reduces signal overlap and enables unambiguous signal assignment and analyses of protein dynamics and protein-ligand binding for large molecular weight protein, transmembrane proteins, and intrinsically disordered proteins.

Methyl group-specific protonation and ^13^C-incorporation of target proteins with uniform deuteration of background protons is advantageous due to the following beneficial NMR characteristics. The intensity of the ^1^H-^13^C signal of a methyl group is approximately three-fold stronger than that of a methine group or ^1^H-^15^N signal of the amide group [82]. Methyl-TROSY-based NMR techniques are essential for inter-molecular interaction studies for large molecular weight and/or membrane proteins [83,84,85,86,87,88,89,90,91,92].

In some cases, assessing ligand binding by measuring simple 1D ^1^H-NMR spectra of methyl groups is possible. Use of highly sensitive and well-resolved methyl group signals for protein-observed ligand binding experiments enables NMR measurements with lower concentration target protein, providing benefits of experimental effectiveness and the possibility of identifying lower affinity hits.

**Figure 4:**
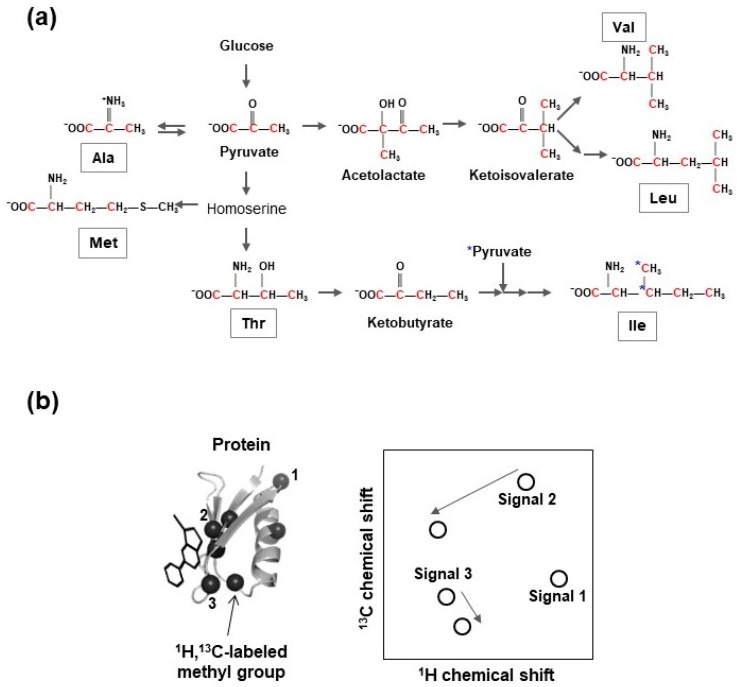
(**a**) Schematic diagram of amino acid biosynthesis and methyl group-specific ^13^C-labeling. Ketobutyrate and ketoisovalerate, precursors of isoleucine and valine/leucine, respectively, are utilized for methyl group-selective ^13^C-labeling of isoleucine, valine, and leucine residues. The red colored carbons are from same origin in metabolism. The blue asterisks denoted on the isoleucine indicate the carbons from pyruvate; (**b**) Chemical shift perturbation method as a protein-based NMR approaches for SBDD studies. Black spheres on the protein, represented with ribbon diagram, indicate position of ^13^C-labeled methyl groups. Ligand is represented with stick diagram. Numbers on the spheres correspond to each ^1^H-^13^C correlation NMR signal on the right panel. Chemical shift perturbation induced by interaction with ligand is indicated by gray arrows.

Isoleucine, leucine, valine (ILV)-selective methyl group ^13^C-labeling, with a uniformly deuterated background, supplies site-specific ^13^C-enriched precursors of ILV, such as 2-keto-3-[^2^H_2_],4-[^13^CH_3_]-butyrate (for ^13^C-labeling of the δ1 methyl group of isoleucine) [83], 2-keto-3-[^2^H]-[^13^CH_3_]_2_-isovalerate (for ^13^C-labeling of all methyl groups of leucine and valine) [84], 2-keto-3-[^2^H]-[^13^CH_3_,^12^C_2_H_3_]-isovalerate (for ^13^C-labeling of either prochiral methyl group of leucine and valine) [85], or ^13^C,^15^N-labeled ILV amino acids, into cell cultivation media prior to the induction of heterologous protein expression (Figure 4). Furthermore, stereospecific ^13^C-labeling of methyl groups of leucine and valine can be accomplished using 2-acetolactate as the amino acid precursor [93].

Using the aforementioned labeling schemes, ^13^C can be incorporated into both leucine and valine. Individual ^13^C-labeling of every amino acid and γ2-methyl group-specific ^13^C-labeling of isoleucine have been developed [92,94,95]. The ε-methyl group-selective ^13^C-incorporation of methionine can be accomplished using ^13^C-labeled methionine or 4-[^13^C]methylthio-2-ketobutyrate as precursor [96,97,98]. Enzymatic and chemical synthesis processes for the generation of 2-[^2^H]-3-[^13^C]-l-alanine and 2,3-[^2^H]_2_-4-[^13^C]-l-threonine have been established [99,100].

Co-application of these precursors and the cocktail of deuterated metabolic intermediates of alanine and threonine, in order to suppress their scrambling to other methyl-containing amino acids, and methyl group-selective ^13^C-labeling of alanine and threonine, have been accomplished [95,99,100,101].

Fractional ^13^C-labeling methods were developed mainly using sparsely ^13^C-labeled glucose [102,103], glycerol [104,105], pyruvate [106,107,108], acetate [109], or erythrose [110] as carbon sources. Methyl group-selective ^1^H,^13^C-incorporation of threonine residues was accomplished by applying a fractional ^13^C-labeling scheme using 2-[^13^C] glycerol and NaH^13^CO_3_ [104,108,111]. Several NMR measurement techniques have identified protein-ligand binding sites using amino acid-selective or site-specific isotope enriched protein samples [76,77].

#### 3.2.1. ^19^F-Labeled Amino Acid Analog Incorporation

Sequence-specific ^19^F-incorporation into a target protein is accomplished by substituting the desired amino acids with fluorinated analogs [112,113,114,115,116], since the natural abundance of ^19^F is 100%. ^19^F-NMR is a useful alternative to ^1^H-based NMR approaches for the quantitative and qualitative investigation of various structural and functional characteristics of the target protein, such as conformational fluctuation dynamics, timescale of enzymatic reaction turnover, and exchange rates of protein-ligand interactions [54,112,117]. The superior susceptibility of ^19^F chemical shifts against its chemical environment is useful for a protein-based NMR approach in SBDD and for characterization of protein structure formation and thermal stability [54,118].

Fluorinated analogs of tryptophan, tyrosine, phenylalanine, leucine, methionine, histidine, and cysteine, including 5- or 6-fluoro-l-tryptophan, 3-fluoro-l-tyrosine, 3- or 4-fluoro-l-phenylalanine, trifluoromethyl-l-phenylalanine, 2-amino-3-(4-(trifluoromethoxy)phenyl) propanoic acid, 5-fluoro-l-leucine, fluoromethyl-l-methionine, and 2-fluoro-l-histidine are commercially available [112]. Fluorine labeling of the aromatic residues of proteins can be accomplished by allowing those analogs to enter the primary sequence of the desired protein during recombinant gene translation using appropriate protein expression systems [119,120,121,122].

For the fluorination of aromatic amino acids during protein expression, glyphosate, which inhibits de novo aromatic amino acid synthesis by blocking the Shikimate pathway, is added into the cell cultivation medium [121,122]. For the fluorination of tryptophan, indoleacrylic acid, which is an inhibitor of tryptophan synthase, is also added [121,122].

A simple and robust tryptophan fluorination method was reported [120]. In this procedure, 5-fluoloindole, an indole moiety containing fluorine, is added into the cell culture medium as a precursor of 5-fluoro-l-tryptophan, and no other inhibitor is required. This simple approach is convenient for preliminary or first-trial tryptophan fluorination of proteins and subsequent ^19^F-NMR measurements, although 6-fluoro-labeling of tryptophan residues is less successful than 5-fluoro-labeling with this method [123]. Using a chemical synthesis method, fluorinated unnatural amino acids can be incorporated into a target peptide, and various kinds of fluorinated peptides can be created and used to discover novel and potent peptides as effective PPI modulators [112].

^19^F spin relaxation is mainly driven by chemical shift anisotropy in T_2_ and dipole–dipole interaction with surrounding NMR-active nuclei in T_1_. This dipolar interaction provides heteronuclear ^1^H-^19^F or homonuclear ^19^F-^19^F NOEs possessing useful information about the distance between these nuclei. Relaxation rate analyses of ^19^F spins reveal the time scale of local mobility of the proteins.

^19^F resonance assignments are required when amino acid residues of the target protein are replaced by corresponding fluorinated analogs. Based on the backbone assignments, pulse sequences such as HCCF-COSY can be applied for ^19^F signal assignments [124]. Unambiguous assignment of those ^19^F resonances can be accomplished by site-directed single mutagenesis, if the number of ^19^F signals is limited and the ^19^F spectrum is well dispersed. This results in the disappearance of the ^19^F signal of the corresponding site [121].

Single-position substitution by fluorinated analogs is powerful since ^19^F signal assignment is not required. This substitution is achieved for phenylalanine using a cell-free protein expression system, combining ^19^F unnatural amino acid and an artificial codon, or an *E. coli* expression system using the amber codon, an artificial tRNA, and yeast tRNA synthetase [125,126]. This site-specific ^19^F-labeling approach is strong for in-cell NMR experiments due to its high, sensitivity simplification of spectra, and low background signals [127,128,129,130].

#### 3.2.2. Isotope-Enriched Unnatural Amino Acid Incorporation

In a similar process to the ^19^F-labeling of aromatic residues as described above, ^13^C/^15^N-labeled unnatural amino acids, such as *p*-methoxy-l-phenylalanine (*p*-OMe-l-Phe) or *o*-nitrobenzyl-l-tyrosine (*o*-NB-l-Tyr), are incorporated into a desired single site of target proteins [131,132].

#### 3.2.3. SAIL

The stereo-array isotope labeling (SAIL) technology is an ideal strategy for site-specific NMR analyses [133]. SAIL amino acids are introduced into target proteins using cell-free protein synthesis. Some SAIL amino acids can be efficiently incorporated into target protein using *E. coli* protein expression systems [134,135]. Although SAIL amino acids were surprisingly expensive before, now the minimum cost is 2000 USD/protein (Taiyo Nippon Sanso, Tokyo, Japan).

### 3.3. Isotope Labeling of Protein by Post-Translational Chemical Modification

In this section, we review post-translational chemical modification approaches for isotope labeling of purified target protein, including paramagnetic labeling.

#### 3.3.1. Site-Specific ^19^F-Labeling

A trifluoromethyl (-CF_3_) or trifluoroacetyl (-COCF_3_) derivative is conjugated to the sulfhydryl group of cysteine residues by covalent bond formation [108,136]. The signal intensity of the trifluorine moiety is higher than that of the monofluorinated form. However, the trifluorinated alkyl group has a larger excluded volume than the single fluorine forms. This exclusion may induce undesired changes to conformation, ligand binding mode, and physicochemical characteristics, such as hydrophobicity and solubility of the target protein. Moreover, new intra- and inter-molecular hydrogen bonds could be generated since the fluorine atom can behave as a hydrogen bond acceptor.

Regardless of the number of fluorine atoms present, the structure and function of the target protein should not be significantly affected by ^19^F-incorporation. In this regard, the execution and analysis of appropriate assays and NMR measurements before the ligand binding experiments can be helpful. Single fluorination of aromatic side chains has a relatively small effect on the structure and function of a protein since the van der Waals radius of a fluorine atom is similar to that of a proton [54,137].

#### 3.3.2. Attaching ^13^C-Methyl Groups

^13^C-*S*-methylthiocysteine includes a ^13^C-labeled methyl group covalently conjugated to the free sulfhydryl group of cysteine. The chemical structure of ^13^C-*S*-methylthiocysteine is similar to methyl group-specific ^13^C-enriched methionine, and this chemical modification is useful for very large proteins [138]. As an analogy, ^19^F_3_-*S*-methylthiocycteine may be useful for ^19^F-NMR studies.

A ^1^H/^13^C-enriched methyl group is attached to an ε-amino group of the lysine side chain of unlabeled target protein by a spontaneous reductive methylation reaction [139,140]. This reaction progresses under physiological conditions without marked influence on the structure or physicochemical characteristics of the target protein. The ^1^H-^13^C correlation signal of the methyl group attached to the lysine side chain is highly sensitive, and the signal can be clearly detected, even if the protein concentration is extremely low (sub-micromolar), which is generally difficult to detect using ^1^H-^15^N correlation resonances [140]. This technique helps NMR-based PPI characterization of challenging proteins, such as membrane proteins, where the preparation of sufficient quantities of isotope-labeled sample is difficult due to their low solubility and limited over-expression [140,141].

#### 3.3.3. ^15^N- and ^19^F-Incorporation into Glutamine Side Chain by Protein Transglutaminase

**Figure 5:**
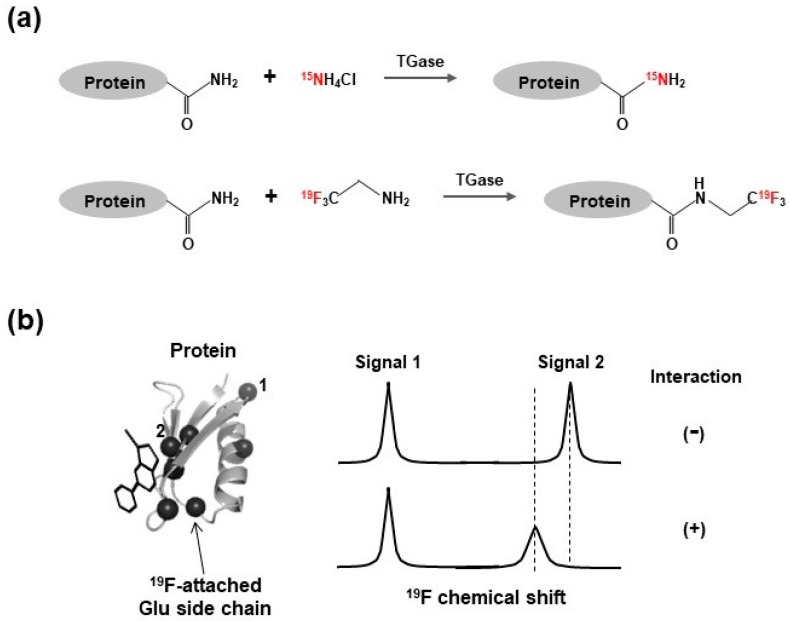
(**a**) Schematic diagrams of enzymatic ^15^N- and ^19^F-labeling of γ-carboxyamide groups of glutamine residues of target protein. ^15^N- and ^19^F-labeled atoms are colored in red; (**b**) Black spheres on the protein indicate position of ^19^F-labeled γ-carboxyamide groups of glutamine residues. Numbers correspond to each ^19^F 1D NMR signal on the right panel. Chemical shift changes and signal broadening are observed upon interaction with ligand.

The ^1^H-^15^N correlation signal of the side chain carboxyamide group of glutamine and asparagine residues is useful as an NMR probe, since it is sharper than that of the backbone amide signals and is applicable to large molecular weight proteins. For the γ-carboxyamide group of the glutamine residue, enzymatic ^15^N-incorporation is achieved using recombinant protein transglutaminase (TGase) [142,143]. The TGase catalyzes the chemical replacement of the γ-carboxyamide group with free ammonium ions, under mild reaction conditions without structural changes, undesired degradation, or precipitation. Therefore, if the ammonium ions were enriched with ^15^N, the result is site-specific ^15^N-incorporation into the glutamine side chain of target proteins (Figure 5a). This method is applicable to ^19^F-labeling of proteins and its ^19^F-NMR analysis as shown in Figure 5 [144].

#### 3.3.4. Segmental Isotope Labeling

Segmental isotope labeling is typically applied to multi-domain proteins for isotope enrichment of only the desired domain, while the other domains remain in an NMR-invisible form [48,145,146]. The segmental labeling is commonly accomplished using intein or Sortase A [147,148,149] by in vitro ligation of two or more recombinant proteins, where one is isotopically enriched, and the others remain unlabeled. This technology can be applied to improve the solubility and NMR spectrum of target proteins by linking NMR-visible target protein and NMR-invisible solubility enhancement tags, such as GB1 [150,151]. Due to technological developments, over-expression of segmental isotope-labeled multi-protein complexes using *E. coli* expression systems has been developed, such as LEGO-NMR (label, express, and generate oligomers for NMR) technology [48,145,152,153].

#### 3.3.5. Paramagnetic-Labeling

Spin-label reagents, such as MTSL ((1-Oxyl-2,2,5,5-tetramethyl-∆3-pyrroline-3-methyl) methanethiosulfonate) or TEMPO (2,2,6,6-tetramethylpiperidine-1-oxyl), possessing stable free radicals on nitroxide, lanthanide ion chelators, and lanthanide ion binding peptide, can be chemically anchored to desired sites on target proteins [25,154,155,156]. If the targets are calcium/magnesium ion binding proteins or metalloproteins, the calcium/magnesium or metal-binding centers can be used for lanthanide immobilization. Typically, one site on an amino acid side chain on the surface of the target protein, which should be proximal to the ligand binding site but not interfere with ligand binding, is chemically modified by these paramagnetic reagents. Side chains of cysteine, tyrosine, and lysine residues are frequently used for chemical modification [25,154,155,156].

The free sulfhydryl group of the cysteine side chain is widely used for paramagnetic labeling due to its high and specific chemical reactivity under mild solution conditions. Spin-label reagents possessing a sulfhydryl group and a maleimide group can be readily covalently attached to surface-exposed sulfhydryl groups of target proteins. If no cysteine residues exist in the target protein, a cysteine residue is introduced at the desired site of the target protein by site-directed mutagenesis. In these cases, all cysteine residues, except for the desired sites, must be substituted with non-cysteine residues, such as serine or threonine [29].

Two or more solvent-exposed cysteine residues with appropriate thiol–thiol distances are necessary when performing a two-site immobilization of a paramagnetic regent, such as the EDTA-based lanthanide-chelating tag Caged Lanthanide NMR Probes (CLaNPs), in an intra-molecular thiol–thiol bridging manner [157]. The target protein tends to aggregate with increasing number of cysteine residues, making the use of this approach difficult.

Saio and co-workers developed a two-point anchoring method, using the lanthanide-binding peptide tag (LBT) [158], by extending the two-site cysteine bridging approach described above. In their method, originally modified LBT, which has one free cysteine at its N-terminus, was over-expressed and simultaneously fused to the N-terminus of the target protein. The sulfhydryl group of the N-terminal cysteine residue on the LBT then spontaneously forms a disulfide bond with the thiol group of another cysteine residue present on the surface of the target protein, like a closing of handcuffs, under non-reducing conditions. This provides dual-point immobilized less-mobile LBT, and leads to stronger PCS and more accurate protein structure determination, compared to that of traditional single-point anchored LBT [158]. They determined the tertiary structure of a protein-drug complex using this approach [159], and developed NMR-based SBDD systems where PRE and PCS were used for fragment screening and for tertiary structure determination of target protein-hit fragment complexes, respectively [28].

Spin-labeling and PRE techniques can be used to seek off-target drug-binding sites on target proteins. When a hit fragment for the first pharmacophore can be identified, a spin-labeled fragment and its complex with the target protein are prepared. Next, fragment screening is performed again to seek new fragments that bind to the target protein/spin-labeled fragment complex. If a new fragment is bound to a second binding site on the protein, and is proximal to the first-ligand binding site, ^1^H or ^19^F resonances of the new fragment can be satisfied by the spin-labeled fragment bound to first-ligand binding site [160]. This technique is powerful in fragment-linking approaches such as SAR-by-NMR.

## 4. Concluding Remarks

Pharmaceutical compounds are molecules that strongly interact with target biomolecules with high specificity, and finely promote or inhibit biological activity or functions of the target biomolecules. Structure-based drug discovery or development studies are rational strategies that can be used to identify and develop new pharmaceuticals.

In this review article, we outlined advances and applications of NMR-based methods aimed at SBDD, and the necessary sample preparation required. PPI will likely be of future importance as a drug target, and basic protein NMR studies used for the identification of hot spots of PPIs and related physicochemical understanding, especially in terms of energetics, should assist in the development of concrete guidelines about the kinds of compounds we need to study for drug development. In that sense, a theoretical and practical understanding of the behavior and dynamics of spins in NMR experiments of spin relaxation is of fundamental importance for fine SBDD that uses changes in conformational dynamics or biological function of the target protein caused by protein–protein or protein–ligand interactions [36,161].

For effective PPI modulators, peptide-like compounds have promising potential since they can mimic or complement the structural and electrostatic environment of the complex PPI interface. Methodologies for the preparation of NMR-oriented peptide libraries, and NMR-based drug development from peptides by NMR measurements of cross-correlated relaxation (CCR) of the peptidic ligands, have been developed by Takahashi et al. [46,162,163,164]. The design of novel lead compounds that mimic pharmacophores of the PPI modulator peptide is one potential strategy that can be used [65,165].

Especially at the stages of hit compound validation and individual lead optimization, co-crystallization of target protein-hit complex, and subsequent X-ray crystallographic analysis is the most robust approach that provides a plethora of information to boost the drug optimization study. Continuous improvements and fine-tuning of NMR-based approaches using the advantages of the methodology to collect tertiary molecular coordinate data of protein-ligand complexes, with higher performance levels compared to X-ray crystallography, are anticipated, especially in efforts to address challenging issues, such as the difficulty in achieving co-crystallization with sufficient quality due to molecular fluctuation of target protein, or pharmaceutical immaturity of the hit fragment.

Further development and optimization of the SBDD methodology can be achieved using the various effective experimental methods to improve the system’s adaptability to individual challenging cases and in every research stage.
